# Deep learning models across the range of skin disease

**DOI:** 10.1038/s41746-024-01033-8

**Published:** 2024-02-10

**Authors:** Kaushik P. Venkatesh, Marium M. Raza, Grace Nickel, Serena Wang, Joseph C. Kvedar

**Affiliations:** Harvard Medical School, Boston, MA USA

**Keywords:** Diagnosis, Medical imaging, Health policy

## Abstract

We explore the evolving landscape of diagnostic artificial intelligence (AI) in dermatology, particularly focusing on deep learning models for a wide array of skin diseases beyond skin cancer. We critically analyze the current state of AI in dermatology, its potential in enhancing diagnostic accuracy, and the challenges it faces in terms of bias, applicability, and therapeutic recommendations.

A systematic review by Choy et al. of 64 deep learning models reveals their high diagnostic accuracy for common skin diseases such as acne, psoriasis, eczema, and rosacea. Some models not only diagnose but also assess disease severity. These increasingly accurate models, though mostly still in research and development, offer opportunity for AI-assisted diagnosis to improve access in face of dermatologist shortages and long wait times; the ability to assess disease severity can build on diagnosis-only outputs to inform treatment decisions and patient self-management. Primary care, given assuring sentiments from providers, presents a particularly apt setting for application of dermatologist-trained models. However, the review also highlights significant challenges, including the need for further refinement in complex diseases, concerns about model bias, and the lack of standardization and diversity in training data. Regulators and providers should implement evaluation criteria for approval and adoption that prioritize applicability and inform decisions on where to best implement these novel technologies.

## Main text

Artificial intelligence (AI) diagnosis in dermatology has moved beyond skin cancer alone to a wide range of common skin diseases - offering exciting new horizons for dermatology care. To date, the FDA has yet to approve an AI device for dermatology diagnosis or treatment^1^. With teledermatology burgeoning during the COVID-19 pandemic, new databanks of skin images have become broadly available to train models^2^.

AI first entered dermatology in the context of Stanford’s landmark deep learning model for skin cancer detection in Nature in 2017^3^. Since then, new models have evolved beyond skin cancer alone—promising significant growth potential for the highly prevalent chronic inflammatory skin diseases, which affect 20–25% of the population wordwide^4–6^. With an array of promising diagnostic models inching closer to the bedside, questions arise for providers and regulators as to how these models should be evaluated and adopted. – particularly as they relate to bias and equity. Moreover, while new AI models has shown proficiency in diagnosing common skin conditions, their ability to navigate the nuances of more complex cases and recommend therapeutic interventions remains a critical area for exploration.

## Promise in diagnosis

Choy et al. conducted a systematic review of 64 non-cancer-related deep learning models for diagnosis and monitoring of 144 different skin diseases^7^. Of these, the most common skin diseases were acne (30), psoriasis (27), eczema (22), rosacea (12), vitiligo (12), and urticaria (8). Most models predicted diagnosis (81%) and the rest, disease severity. Most image datasets (88%) used macroscopic images of skin, hair and nails, with the remainder using dermoscopic images. These image datasets were separated into three types, with varying uses and sizes: training (used to create models; median *n* = 2555), validation (used to evaluate model performance; median *n* = 1032), and testing (final evaluation; median *n* = 331).

Overall, the accuracy of these models was impressive in diagnosing acne (94%), rosacea (94%), eczema (93%) and psoriasis (89%). Accuracy for grading severity was more variable, but still high: psoriasis (93–100%,), eczema (88%), and acne (67–86%). These findings align with those of prior systemic reviews^8^, demonstrating growing evidence of accurate AI diagnostic tools across dermatology – at least for common skin conditions.

Diagnostic assistance from AI models has significant value in increasing access given context of the dermatologist shortage and long wait times (averaging 36 days in the US)^9^. Severity ratings of disease make model outputs more relevant for treatment decisions and patient self-management of chronic disease—a significant advance from diagnosis-only models^10^. However, there remains significant room for improving the nuance of these models to accurately recommend therapeutic changes. Moreover, many of high-accuracy diagnoses (acne, psoriasis, vitiligo) are readily recognizable by most providers; other conditions such as eczema and urticaria may be more difficult, and models that diagnose these and similarly complex diagnoses offer more promise.

The next step in implementation involves identifying the most opportune use cases for such technology. One particularly important application lies in primary care; in one study, 92% of p considered the tested AI dermatology diagnosis model a useful support tool in creating a differential diagnosis, and 60% even considered it useful to determine the final diagnosis^11^. Beyond primary care, other provider groups serving patients with skin conditions should critically analyze whether and when such technology would augment care.

## Pitfalls in applicability

Choy et al. also found nearly ubiquitous bias and applicability concerns; quality assessment with the CLEAR Derm and QUADAS-2 framework^12^ found that 59 studies (92%) had a high risk of bias and 62 (97%) had a high level of applicability concerns. Bias in AI has been a long-standing concern across healthcare. In dermatology, the QUADAS-2 framework and CLEAR Derm guidelines could be useful for future evaluation. Further development of quality assessment tools, with validation in dermatology specifically, is also necessary to ensure that these AI tools do not perpetuate biases.

Moreover, models in the study used varying reference standards (the “correct” diagnoses used to train models), i.e., some used dermatologist-produced diagnoses, while others used PCP-based diagnoses or a combination of both sources of diagnoses. Dermatologists have significantly higher diagnostic accuracy than non-dermatologists given their specialized training^13^, suggesting that dermatologist reference standards should be used in all relevant datasets for the highest quality of care. This choice of providers involved in producing training datasets has ramifications for the care setting in which these technologies will be used. For example, dermatologists may be wary to adopt models trained with PCP-generated data, while PCPs may be more amenable to such a model.

AI has immense potential in increasing access to care, including new data on autonomous AI showing promise in increasing productivity^14^. However, Choy et al. found that only 19% of models reported ethnicity or Fitzpatrick skin type (skin color gradation). Even among those reporting, darker skin types were underrepresented - leaving significant concerns regarding whether these findings are applicable to marginalized populations, who often face the most challenges accessing dermatology care. Skin diversity metrics of training datasets should be mandatory in the academic literature and for product approval. Moreover, regulators and industry should consider requiring validation and testing with diversity-certified datasets, particularly for models trained on private and undisclosed datasets.

## Looking forward

Overall, deep learning models in dermatology have promising accuracy in diagnosis and severity classification among numerous common skin diseases, though they still present limitations in recommending therapy with nuance. Models are further challenged by significant risk of bias, applicability concerns, varying reference standards, and poor diversity representation. As the scope of AI utilization continues to expand, evaluation frameworks are necessary to evaluate bias, standardize dataset training produced by dermatologists, and ensure representation of diverse skin phenotypes. As we usher in this new era of digital dermatology, it is imperative for researchers, clinicians, and policymakers to collaboratively navigate these uncharted waters, ensuring that AI tools are developed and implemented thoughtfully, with an eye towards their ultimate goal: enhancing patient care and outcomes for all.

## References

[1] Health C for D and R. Artificial Intelligence and Machine Learning (AI/ML)-Enabled Medical Devices. *FDA*. Published online October 5, 2022. Accessed October 15, 2023. https://www.fda.gov/medical-devices/software-medical-device-samd/artificial-intelligence-and-machine-learning-aiml-enabled-medical-devices

[2] Wen D (2022). Characteristics of publicly available skin cancer image datasets: a systematic review. Lancet Digit. Health..

[3] Esteva A (2017). Dermatologist-level classification of skin cancer with deep neural networks. Nature..

[4] Li X, Zhao X, Ma H, Xie B (2023). Image analysis and diagnosis of skin diseases - a review. Curr. Med. Imaging..

[5] Jeong HK, Park C, Henao R, Kheterpal M (2023). Deep learning in dermatology: a systematic review of current approaches, outcomes, and limitations. JID Innov..

[6] Ujiie H (2022). Unmet medical needs in chronic, non-communicable inflammatory skin diseases. Front. Med..

[7] Choy SP (2023). Systematic review of deep learning image analyses for the diagnosis and monitoring of skin disease. npj Digit Med..

[8] Kassem MA, Hosny KM, Damaševičius R, Eltoukhy MM (2021). Machine learning and deep learning methods for skin lesion classification and diagnosis: a systematic review. Diagnostics.

[9] Uhlenhake E, Brodell R, Mostow E (2009). The dermatology work force: a focus on urban versus rural wait times. J. Am. Acad. Dermatol..

[10] Huang K (2023). Artificial intelligence-based psoriasis severity assessment: real-world study and application. J. Med. Internet Res..

[11] Escalé-Besa A (2023). Exploring the potential of artificial intelligence in improving skin lesion diagnosis in primary care. Sci. Rep..

[12] Sounderajah V (2021). A quality assessment tool for artificial intelligence-centered diagnostic test accuracy studies: QUADAS-AI. Nat. Med..

[13] Chen SC, Bravata DM, Weil E, Olkin I (2001). A comparison of dermatologists’ and primary care physicians’ accuracy in diagnosing melanoma: a systematic review. Archives Dermatol..

[14] Abramoff MD (2023). Autonomous artificial intelligence increases real-world specialist clinic productivity in a cluster-randomized trial. npj Digit Med..

